# A posterior-only approach for ankylosing spondylitis (AS) with thoracolumbar pseudoarthrosis: a clinical retrospective study

**DOI:** 10.1186/s12891-020-03402-2

**Published:** 2020-06-11

**Authors:** Jianqiang Kou, Jianwei Guo, Xiangli Ji, Xiaojie Tang, Xiangyun Liu, Yuanliang Sun, Xiujun Zheng, Yingzhen Wang

**Affiliations:** 1 Department of Orthopedics, The Affiliated Hospital of Qingdao University, 16 Jiangsu Road, Qingdao, 266003 Shandong Province People’s Republic of China; 2 Department of Intensive Care Unit, Qilu Hospital of Shandong University (Qingdao), 758 Hefei Road, Qingdao, 266035 Shandong Province People’s Republic of China; 3 Department of Orthopedics, Yantai Affiliated Hospital of Binzhou Medical University, 717 Jinbu Street, Muping District, Yantai, 264000 Shandong Province People’s Republic of China

**Keywords:** Ankylosing spondylitis, Pseudoarthrosis, Posterior approach, Anterior fusion, Osteotomy

## Abstract

**Background:**

Surgical treatment has been recommended by most surgeons to treat pseudarthrosis in ankylosing spondylitis (AS). However, there is still some debate on the necessity of anterior fusion. There is very limited literature on the treatment and surgical outcomes of thoracolumbar pseudarthrosis in AS patients treated through a posterior-only approach.

**Methods:**

From January 1, 2012 to December 31, 2017, a total of 42 cases diagnosed with thoracolumbar pseudarthrosis in AS patients with moderate kyphosis were included in this study. All of the patients received posterior-only kyphosis correction, internal fixation and fusion without anterior fusion, and underwent at least 2 years of follow-up. Clinical and radiographic results and complications were assessed.

**Results:**

All of the patients were followed up for an average of 35.3 months (range, 24–48 months), and they achieved successful bone graft fusion at the pseudarthrosis sites. Satisfactory radiographic changes were achieved in these patients. The Cobb angles of global kyphosis (GK) were corrected from 53.2 ± 5.4 degrees preoperatively to 33.2 ± 4.3 degrees postoperatively, and to 36.1 ± 5.3 degrees at the latest follow-up. The Cobb angles of local kyphosis (LK) were corrected from 43.3 ± 4.6 degrees preoperatively to 26.8 ± 3.3 degrees postoperatively, and to 28.2 ± 3.6 degrees at the latest follow-up. The mean sagittal vertical axis (SVA) were corrected from 7.6 ± 4.2 cm preoperatively to 4.3 ± 2.1 cm postoperatively, and to 4.8 ± 2.3 cm at the latest follow-up. No serious neurological complication or deep wound infection was found in these 42 patients.

**Conclusion:**

Posterior-only kyphosis correction and fixation without anterior fusion can achieve excellent bone fusion and satisfactory improvement in AS patients with thoracolumbar pseudarthrosis. This method may be a good choice for treating thoracolumbar pseudarthrosis in AS patients with moderate kyphosis.

## Background

Ankylosing spondylitis (AS) is characterized by progressive rheumatic inflammation, which mainly affects the spine and sacroiliac joints and causes ossification of the ligaments of the spinal column, intervertebral disc, and zygapophyseal joints, called “bamboo spine” [1–3]. Inflammation and ossification of the ligaments can cause osteopenia and spinal rigidity, making the patient prone to sustaining spinal fracture, even after minor trauma [4, 5]. Spinal fracture in AS patients after minor trauma may not attract any attention and may cause a delayed diagnosis of spinal fracture, leading to pseudarthrosis at the destructive site [6, 7]. Pseudarthrosis may cause kyphosis deformity and severe back pain, even neurologic sequelae [8, 9].

Pseudarthrosis in AS patients is highly unstable because the lesion can involve both the anterior and posterior columns of the spine [10]. Conservative treatment is not successful in AS patients with pseudarthrosis, even after a long treatment course [10]. Appropriate surgical procedure may be a good choice for patients who fail to improve after conservative treatment.

However, the optimal surgical treatment remains controversial. Anterior, posterior and combined approaches have been used for the treatment of pseudarthrosis and correction of the kyphotic deformity in patients with AS [11–13]. Since Chang first reported posterior-only correction and fixation without anterior fusion for treatment of AS patients with pseudarthrosis and kyphotic deformity [14], there is still very limited literature on this method. The aim of this study is to evaluate the safety and effectiveness of this method and to show the salient features of this method.

## Methods

After approval by the Institutional Review Board at our hospital, 42 patients diagnosed with AS in the affiliated hospital of Qingdao University and Yantai Hospital of Binzhou Medical College with progressive kyphosis and potential instability at the sites of thoracolumbar pseudarthrosis from January 1, 2012 to December 31, 2017 were included in this study. All of the patients were treated through a posterior-only approach without anterior fusion. The 42 cases included 33 males and 9 females, with the age of 46.3 ± 12.5 years. All of the patients met the inclusion criteria and exclusion criteria. Inclusion criteria were defined as follows: 1) diagnosed with AS, 2) recurrent back pain, especially by positional changes, 3) progressive kyphosis and computed tomography- (CT) or magnetic resonance imaging- (MRI) confirmed thoracolumbar pseudarthrosis at the sites of pathologic fracture, 4) moderate kyphosis, and the Cobb angle of global kyphosis less than 60 degrees, and 5) at least 2 years of regular follow-up. Exclusion criteria included the following: 1) acute spine fracture with or without AS, 2) neurological deficits at admission, 3) multiple concomitant fractures, 4) pseudarthrosis located at the cervical vertebrae, 5) severe kyphosis and the Cobb angle of global kyphosis more than 60 degrees, and 6) follow-up period of less than 2 years.

All of the patients received radiographic examination before surgery, including X-ray, CT, and MRI. Among these 42 patients, 30 patients were found to have discovertebral destructive lesions and 12 patients were found to have transvertebral destructive lesions. These lesions not only involved the discovertebral or transvertebral sites, but they also affected the posterior structure of the spine, causing mechanical instability and progressive kyphosis.

Standing lateral radiographs were obtained preoperatively, postoperatively, and at the latest follow-up. Radiographic parameters, including global kyphosis (GK), local kyphosis (LK), and sagittal vertical axis (SVA), were measured to evaluate the sagittal alignment changes. GK was defined as the angle between the upper endplate of the maximally tilted upper end vertebra and the lower endplate of the maximally tilted lower end vertebra. LK was defined as the angle between the upper endplate of the vertebra cephalad to the pseudarthrosis site and the lower endplate of the vertebra caudal to the pseudarthrosis site. SVA was defined as the distance between the C7 plumb line and the posterior superior corner of S1.

CT was used to evaluate the bone fusion during follow-up. Bone fusion was defined as the presence of bony trabeculae across the previous pseudarthrosis site. Besides, clinical outcomes were also assessed by the visual analogue scale (VAS) and Oswestry Disability Index (ODI) preoperatively and at the latest follow-up.

### Surgical technique

**Fig. 1:**
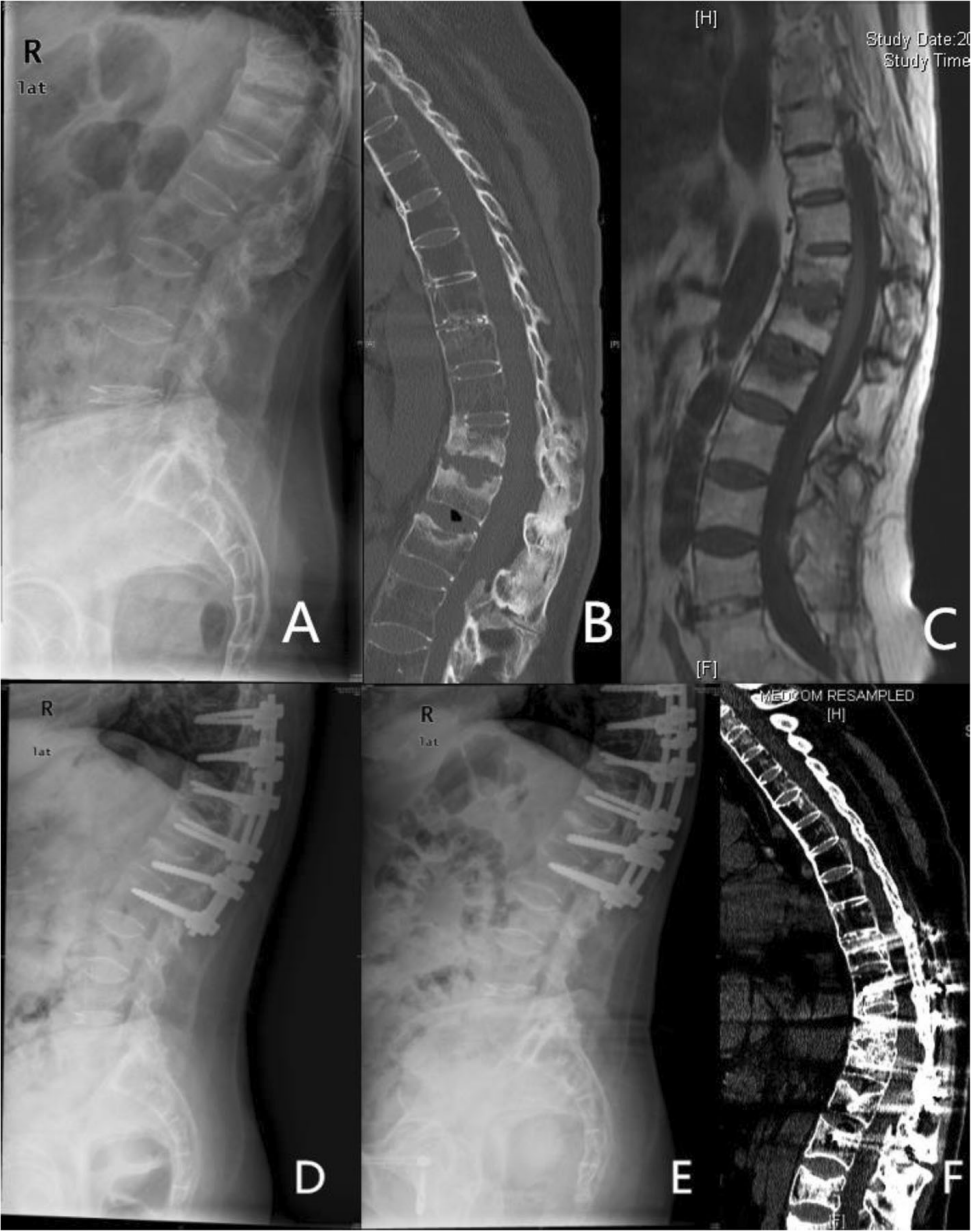
A 61- year old female suffered from progressive kyphosis and mechanical pain for 1 year. Sagittal X-ray of the lumbar spine (**a**) showed a discovertebral destructive lesions at T10/11. A sagittal reconstructed CT image and MRI (**b** and **c**) showed central osteolysis and surrounding sclerotic endplate at T10/11. Sagittal X ray of lumbar spine after the operation (**d**) showed good internal fixation and kyphosis correction without anterior fusion. Sagittal X ray of lumbar spine (**e**) and sagittal reconstructed CT image (**f**) at 2-year follow-up confirmed good fusion of the pseudarthrosis at T10/11

All of the patients were treated by posterior kyphosis correction and internal fixation with pedicle screw instrumentation. Somatosensory-evoked potentials (SEPs) and motor-evoked potentials (MEPs) were recorded during the surgery. The patients were placed in a prone position on a special V-folding operating bed, accommodating the kyphotic spine and avoiding further injury to the spinal cord. The fusion level usually included at least 2–3 segments upper and lower than the pseudarthrosis sites, based on the osteoporosis conditions of patients. After internal fixation with pedicle screws, kyphosis correction was performed with the compression force on the pedicle screws above and below the pseudarthrosis site and extension of the folding operation bed, using the posterior marginal elements of the pseudarthrosis site as the fulcrum (Fig. 1). SEPs and MEPs were monitored during kyphosis correction, thus avoiding neurologic impairment. For those whose MRI or CT showed spinal canal encroachment, laminectomy at the site of pseudarthrosis was performed to ensure the absence of compression on the spinal dural sac. Brace protection was mandatory for at least 3 months postoperatively to confirm bone graft fusion. All of the patients were followed up at 3, 6, and 12 months after the surgery and then at a yearly interval. Spinal X-ray radiography was performed at every follow-up to record the condition of instrumentation and CT was performed at the 1-year follow-up to assess the condition of bone fusion at the pseudarthrosis site.

### Statistical analysis

All of the clinical and radiographic data were reported as means and standard deviation (SD). Two-way repeated ANOVA with post hoc analysis was used to compare the preoperative, postoperative and the latest follow-up clinical and radiographic data. Statistical analysis was performed with SPSS 17.0 (SPSS Inc., Chicago, IL, USA), and *P* ≤ 0.05 was defined as indicative of statistical significance.

## Results

All of the patients, including 33 males and 9 females, received posterior-only kyphosis correction and fixation without anterior fusion. These patients were followed up for an average of 35.3 months (range, 24–48 months). The mean operative time was 113.4 ± 22.6 min, and the mean blood loss was 121.4 ± 22.6 ml. The VAS value improved from 6.23 ± 1.05 cm preoperatively to 2.13 ± 1.13 cm postoperatively, and to 1.54 ± 0.72 cm at the latest follow-up. The ODI value improved from 76.8 ± 7.3% preoperatively to 26.4 ± 8.2% postoperatively, and to 28.4 ± 7.8% at the latest follow-up.

All of the patients achieved successful bone graft fusion at the pseudarthrosis sites. Significant radiographic changes were achieved in all of the patients. The Cobb angles of GK corrected from 53.2 ± 5.4 degrees preoperatively to 33.2 ± 4.3 degrees postoperatively, and to 36.1 ± 5.3 degrees at the latest follow-up. The Cobb angles of LK corrected from 43.3 ± 4.6 degrees preoperatively to 26.8 ± 3.3 degrees postoperatively, and to 28.2 ± 3.6 degrees at the latest follow-up. The mean SVA corrected from 7.6 ± 4.2 cm preoperatively to 4.3 ± 2.1 cm postoperatively, and to 4.8 ± 2.3 cm at the latest follow-up (Table 1).

**Table 1:** Summary of radiological and clinical data of 42 AS patients with pseudarthrosis

	Pre-operation	Post-operation	the latest follow-up
GK	53.2 ± 5.4	33.2 ± 4.3*^1^	36.1 ± 5.3^†1^
LK	43.3 ± 4.6	26.8 ± 3.3*^2^	28.2 ± 3.6^†2^
SVA	7.6 ± 4.2	4.3 ± 2.1*^3^	4.8 ± 2.3^†3^
VAS (cm)	6.23 ± 1.05	2.13 ± 1.13^*4^	1.54 ± 0.72^†4^
ODI (%)	76.8 ± 7.3	26.4 ± 8.2^*5^	28.4 ± 7.8^†5^

*GK* global kyphosis, *LK* local kyphosis, *SVA* sagittal vertical axis

Two-way repeated ANOVA with post hoc analysis was used to compare the preoperative, postoperative and the latest follow-up radiographic data. There were significant differences between preoperative and postoperative, postoperative and the latest follow-up in radiographic data for *P* value less than 0.05 (* between preoperative and postoperative, † between preoperative and the latest follow-up). ^*1^P = 0.019, ^*2^P = 0.008, ^*3^P = 0.006, ^*4^P = 0.000, ^*5^P = 0.000, ^†1^P = 0.026, ^†2^P = 0.017, ^†3^P = 0.012, ^†4^P = 0.000, ^†5^P = 0.000,

No serious neurological complication or deep wound infection was found in these 42 patients. Two patients had symptoms of cerebrospinal fluid leakage, and one patient had delayed wound union. There was no instrument-related complication at the final follow-up.

## Discussion

Because of vertebral osteoporosis and deformed spine alignment, AS patients are prone to sustain spinal injuries even after low-energy trauma or without any obvious cause [4, 5]. Pseudarthrosis will occur when these fractures lose recognition and treatment. This phenomenon was first described by Andersson in 1937 and is known as Andersson lesions (ALs) [15]. AS patients with pseudarthrosis may suffer from persistent or mechanical back pain, progressive neurologic deficit, aggravating kyphosis deformity, and sagittal imbalance either in isolation or in combination [8, 9]. Previous studies have reported that 1.5% to more than 28% of AS patients might experience this type of complications, located in the vertebral or discovertebral destructive lesions [10].

The etiology of ALs in AS patients has not yet been clarified. Histological examination of ALs has revealed the presence of hypo-vascular fibrous tissue and mild inflammatory changes with infiltration of plasma cells, lymphocytes, and macrophages in the lesions, revealing that inflammation or infection may be one of the causes of pseudarthrosis [16, 17]. Additionally, a mechanical factor may play an important role in the formation of ALs [16, 17]. Pseudarthrosis may form when an acute fracture occurs through an already fused segment or repeated stress causes a fatigue fracture in the ankylosed spine without treatment. Most researchers believe that both inflammatory factors and mechanical factors are crucial for the formation of ALs [16, 17].

Management of pseudarthrosis in ALs is really complex. Generally nonsurgical treatment of spine injuries in ALs is only recommended for nondisplaced and clinically stable deformity. However, secondary fracture displacement in injuries, which are more unstable than expected, and patients’ intolerance to bracing make these conservative treatments difficult to perform. Altered biomechanics and alignment of the spine in ALs make these injuries highly unstable [18, 19]. Besides, patients managed with nonsurgical treatment have a higher risk of developing pulmonary complications, thromboembolism, and decubitus ulcers after bed rest [20, 21]. Lukasiewicz et al. reported that 49.9% of patients underwent instrumented fusion surgery in a retrospective cohort study of 939 patients managed with nonsurgical treatment [22]. Nonsurgical treatment of spine injuries in ALs may not be a good choice.

Surgical treatment of spinal fractures in ALs was advocated by many surgeons. Different surgical approaches have been used to treat this type of spinal deformity, including the posterior approach, anterior approach, and combined approach. The anterior-only approach was limited because pseudarthrosis in ALs usually involved anterior and posterior columns and had osteoporosis, making anterior-alone fixation unable to provide adequate screw purchase and posterior stabilization [23]. Both the posterior approach and the combined approach have achieved posterior stabilization and bone fusion during the follow-up, and they are recommended by most surgeons. However, there is a debate on the need of the anterior approach for pseudarthrosis curettage and bone graft [16, 24, 25]. Anterior debridement and bone graft fusion have been proved to achieve good results. Zhang et al. suggested posterior osteotomy through the pathological fracture gap and fixation, and they achieved satisfactory correction of kyphosis and bone graft fusion [26]. However, this method increased the operation time and blood loss, and it might also increase the rate of surgical complications.

AS has a strong ability for bone reunion [13, 14]. All of the soft tissues surrounding the spine, including the ligaments, discs, and joints, will gradually fuse and the unstable spine fracture will become stable. Chang et al. suggested that anterior fusion was not necessary and posterior correction and fixation without anterior fusion for pseudarthrosis with kyphosis deformity in AS patients can achieve good kyphosis correction and excellent bone fusion [14]. In our study, anterior fusion was not performed and successful kyphosis correction and excellent bone fusion were achieved. This method requires two procedures. First, SEPs and MEPs should be performed to monitor the spinal cord signal during the operation; thus, avoiding neurological impairment. Second, kyphosis must be corrected gradually and gently. In patients whose MRI or CT shows spinal canal encroachment, laminectomy at the site of pseudarthrosis should be performed to ensure that there is no compression of the spinal dural sac, thus avoiding sagittal translation of posterior marginal elements at the pseudarthrosis site during kyphosis correction.

We think that posterior-only kyphosis correction and fixation without anterior fusion is a good choice for the treatment of pseudarthrosis with moderate kyphosis deformity in AS patients. However, this method has its own limitations. First, the sample size of patients included in this study is limited and more patients in multiple medical centers are needed in the future. Second, this study is not a case-control study. Third, this article only reported the results of the posterior-only method and it did not compare the findings with the results of the posterior method with anterior fusion and the combination of anterior and posterior methods. Further, this article only reported the results in AS patients with moderate kyphosis and no neurologic defects. Patients with severe kyphosis and neurologic defects should be included in further studies. Longer follow-up and multi-center studies are needed in the future to accurately evaluate the safety, effectiveness, and complications of this method.

## Conclusion

Based on the excellent reunion ability of AS, anterior fusion may not be necessary for pseudarthrosis in AS patients. Posterior-only kyphosis correction and fixation without anterior fusion or osteotomy can achieve obvious kyphosis correction, excellent bone fusion, and satisfactory improvement in back pain. This method may be a good choice for pseudarthrosis in AS patients with moderate kyphosis. Further research is needed to investigate the safety and effectiveness of this method in a large study population with a longer follow-up.

## Data Availability

The data used and analyzed during the current study are available in anonymized form from the corresponding author on reasonable request.

## Abbreviations

AS: ankylosing spondylitis
CT: computed tomography
MRI: magnetic resonance imaging
LK: local kyphosis
GK: global kyphosis
SVA: sagittal vertical axis
VAS: visual analogue scale
ODI: Oswestry Disability Index
SEP: somatosensory-evoked potentials
MEP: motor-evoked potentials
ALs: Andersson lesions

## References

[1] McGonagle D, Gibbon W, Emery P (1998). Classification of inflammatory arthritis by enthesitis. Lancet.

[2] Feldtkeller E, Vosse D, Geusens P (2006). Prevalence and annual incidence of vertebral fractures in patients with ankylosing spondylitis. Rheumatol Int.

[3] Briot K, Roux C (2015). Inflammation, bone loss and fracture risk in spondyloarthritis. RMD Open.

[4] Mulpuri K, Jawadi A, Perdios A (2007). Outcome analysis of chance fractures of the skeletally immature spine. Spine (Phila Pa 1976).

[5] Lems WF (2007). Clinical relevance of vertebral fractures. Ann Rheum Dis.

[6] DiIorio G, Sundaram M (1990). Radiologic case study. Fracture with pseudarthrosis in ankylosing spondylitis. Orthopedics.

[7] Pastershank SP, Resnick D (1980). Pseudoarthrosis in ankylosing spondylitis. J Can Assoc Radiol.

[8] Pettersson T, Laasonen L, Leirisalo-Repo M (1996). Spinal pseudoarthrosis complicating ankylosing spondylitis: a report of two patients. Br J Rheumatol.

[9] Good AE, Keller TS, Weatherbee L (1982). Spinal cord block with a destructive lesion of the dorsal spine in ankylosing spondylitis. Arthritis Rheum.

[10] Rustagi T, Drazin D, Oner C (2017). Fractures in spinal Ankylosing disorders: a narrative review of disease and injury types, treatment techniques, and outcomes. J Orthop Trauma.

[11] Kim KT, Jo DJ, Lee SH (2012). Does it need to perform anterior column support after smith-Petersen osteotomy for ankylosing spondylitis?. Eur Spine J.

[12] Ravinsky RA, Ouellet JA, Brodt ED (2013). Vertebral osteotomies in Ankylosing spondylitis-comparison of outcomes following closing wedge osteotomy versus opening wedge osteotomy: a systematic review. Evid Based Spine Care J.

[13] Wang T, Wang D, Cong Y (2017). Evaluating a posterior approach for surgical treatment of thoracolumbar Pseudarthrosis in Ankylosing spondylitis. Clin Spine Surg.

[14] Chang KW, Tu MY, Huang HH (2006). Posterior correction and fixation without anterior fusion for pseudoarthrosis with kyphotic deformity in ankylosing spondylitis. Spine (Phila Pa 1976).

[15] Andersson O (1937). Röntgenbilden vid spondylarthritis ankylopoetica.

[16] Cawley MI, Chalmers TM, Kellgren JH (1972). Destructive lesions of vertebral bodies in ankylosing spondylitis. Ann Rheum Dis.

[17] Rasker JJ, Prevo RL, Lanting PJ (1996). Spondylodiscitis in ankylosing spondylitis, inflammation or trauma? A description of six cases. Scand J Rheumatol.

[18] Werner BC, Samartzis D, Shen FH (2016). Spinal fractures in patients with Ankylosing spondylitis: etiology, diagnosis, and management. J Am Acad Orthop Surg.

[19] Lautermann D, Braun J (2002). Ankylosing spondylitis--cardiac manifestations. Clin Exp Rheumatol.

[20] Westerveld LA, van Bemmel JC, Dhert WJ (2014). Clinical outcome after traumatic spinal fractures in patients with ankylosing spinal disorders compared with control patients. Spine J.

[21] Caron T, Bransford R, Nguyen Q (2010). Spine fractures in patients with ankylosing spinal disorders. Spine (Phila Pa 1976).

[22] Lukasiewicz AM, Bohl DD, Varthi AG (2016). Spinal fracture in patients with Ankylosing spondylitis: cohort definition, distribution of injuries, and hospital outcomes. Spine (Phila Pa 1976).

[23] Kouyoumdjian P, Guerin P, Schaelderle C (2012). Fracture of the lower cervical spine in patients with ankylosing spondylitis: retrospective study of 19 cases. Orthop Traumatol Surg Res.

[24] Fang D, Leong JC, Ho EK (1988). Spinal pseudarthrosis in ankylosing spondylitis. Clinicopathological correlation and the results of anterior spinal fusion. J Bone Joint Surg Br.

[25] Yau AC, Chan RN (1974). Stress fracture of the fused lumbo-dorsal spine in ankylosing spondylitis. A report of three cases. J Bone Joint Surg Br.

[26] Zhang H, Zhou Z, Guo C (2016). Treatment of kyphosis in ankylosing spondylitis by osteotomy through the gap of a pathological fracture: a retrospective study. J Orthop Surg Res.

